# Case Report: A case of melanosis coli complicated by pneumatosis intestinalis–induced volvulus

**DOI:** 10.3389/fmed.2025.1660123

**Published:** 2025-10-08

**Authors:** Shuxia Bai, Jiangbo Wang, Tingting Song, Wenjia Sun

**Affiliations:** ^1^Department of Pathology, The People’s Hospital of Boertala Mongolian Autonomous Prefecture, Bole, China; ^2^Department of Pathology, Hubei Cancer Hospital, Tongji Medical College, Huazhong University of Science and Technology, Wuhan, China

**Keywords:** melanosis coli, pneumatosis intestinalis, volvulus, pathophysiology, case report

## Abstract

Melanosis coli (MC) combined with pneumatosis intestinalis (PI) is an exceedingly rare condition. We present the case of an 85-year-old male with a history of chronic constipation, hypertensive heart disease, and type 2 diabetes mellitus. The patient was admitted to the hospital because of an acute sigmoid volvulus with complete intestinal obstruction. After 3 days of conservative treatment, surgical intervention was performed, consisting of sigmoidectomy with volvulus reduction and formation of a descending colostomy. Histopathological examination confirmed MC with PI and associated mucosal necrosis. The patient, unfortunately, developed severe complications and eventually died of hemorrhagic shock and circulatory failure 22 days after surgery. This case explores the potential mechanisms underlying MC complicated by PI, emphasizing the need for increased clinical vigilance in elderly diabetic patients with chronic constipation and pre-existing cardiovascular disease, particularly following the use of anthraquinone-based laxatives and alpha-glucosidase inhibitors (αGIs).

## 1 Introduction

Melanosis coli (MC) is a reversible, non-inflammatory, and non-precancerous lesion characterized by the deposition of lipofuscin in the colonic mucosa. Patients with MC typically exhibit no specific symptoms or signs. The gold standard for diagnosing MC includes endoscopic and histopathological examination. Endoscopic examination reveals diffuse, irregular, dark brown discoloration of the colonic mucosa, which is often observed in the skin of crocodiles or snakes (1). The reported incidence of MC ranges from 0.82 to 1.13% (2). MC is typically associated with prolonged use of anthraquinone-containing laxatives, such as Folium Sennae, Rhei Radix et Rhizome, Semen Cassiae, and Aloe. When these stimulant laxatives traverse the colon, they undergo conversion into active metabolites, which induce damage to colonic epithelial cells and subsequently trigger apoptosis. The resulting apoptotic cells are phagocytosed by macrophages and transformed into brownish lipofuscin pigment, which accumulates within the lamina propria (3).

Pneumatosis intestinalis (PI) is defined as the presence of intramural gas accumulation within the bowel wall, typically identified via endoscopy or abdominal computed tomography (CT). Endoscopically, PI can present as solitary, fused, or irregular beaded forms with smooth surfaces. The lesions are soft when touched with biopsy forceps and begin to deform under compression. The cysts collapse when biopsied, followed by the release of gas without fluid outflow (4). A CT scan is the gold standard for confirming the presence of PI, with typical findings showing linear or circular collections of gas in the bowel wall (5). PI can occur anywhere in the gastrointestinal tract with an estimated incidence of approximately 0.03%. PI can be classified into primary (15%) and secondary forms (85%) (6). The pathogenesis of PI is incompletely understood, although three pathophysiological mechanisms have been proposed: 1. Mechanical theory: Suggests that intraluminal gas penetrates the bowel wall either through mucosal defects or via mesenteric vessels. 2. Pulmonary theory: Proposes that chronic pulmonary conditions may lead to alveolar rupture, causing mediastinal emphysema, with subsequent gas dissection along the aortic and mesenteric vessels into the intestinal wall. 3. Bacterial theory: Postulates that gas-forming bacteria invade the mucosa through structural breaches, colonize the submucosa, and generate intramural gas collections (7). Secondary PI has been associated with numerous underlying conditions, including but not limited to inflammatory bowel disease, intestinal ischemia, bowel necrosis, chronic obstructive pulmonary disease (COPD), COVID-19 infection, immunosuppression, and systemic chemotherapy (5).

Cases of MC complicated by PI have rarely been reported, and the underlying mechanism of their co-occurrence remains unclear. This report analyzes the pathogenesis of MC complicated by PI in a patient presenting with acute sigmoid volvulus and necrosis and further summarizes the experiences and lessons learned from the treatment process of this case, aiming to raise clinical awareness among high-risk populations.

## 2 Case report

**Figure 1:**
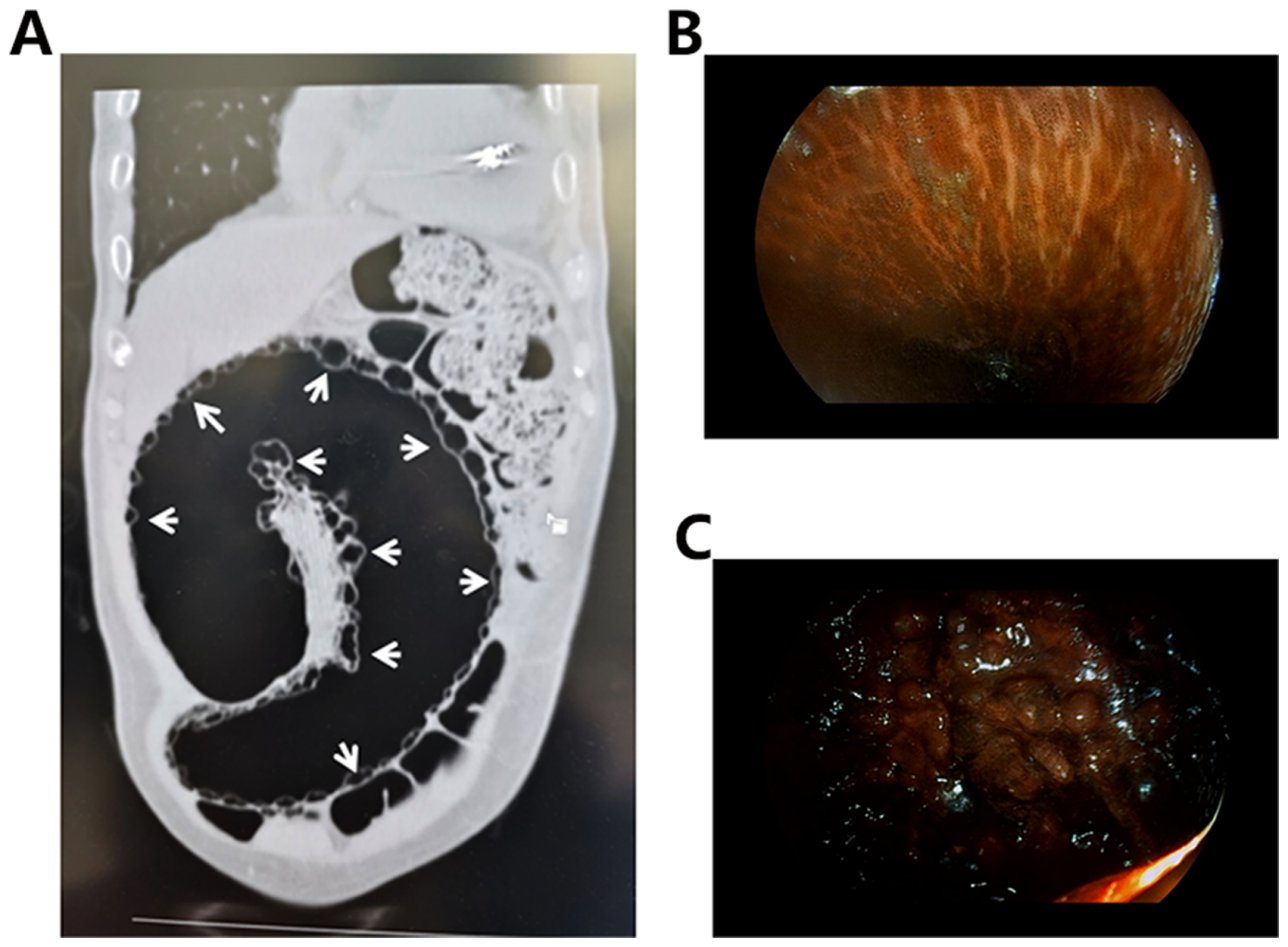
**(A)** Abdominal CT shows a number of grape-like or beaded low-density cystic light transmission areas in the bowel wall (white arrows). **(B)** Endoscopic images of the MC show brown or black pigmentation of the intestinal mucosa, presenting a crocodile-like appearance. **(C)** Endoscopy shows darkening of the intestinal mucosa with ischemic and necrotic changes.

An 85-year-old male presented with a one-year history of intermittent lower abdominal distension and pain, which acutely worsened over the preceding day, accompanied by reduced flatus and defecation. His medical history included chronic constipation for 3 years, managed effectively with Binglang Sixiao Pills (containing Areca Seed, Rhei Radix Et Rhizoma, Pharbitis Seed, Rhizoma Cyperi, and Trogopterus Dung). Additionally, he had a 10-year history of hypertensive heart disease and a six-year history of poorly controlled type 2 diabetes mellitus treated with acarbose and dapagliflozin. On physical examination, he exhibited abdominal distension with diffuse tenderness but no rebound tenderness. Bowel sounds were present at 4/min, accompanied by audible gurgling. Routine blood tests revealed leukocytosis and neutrophilia, whereas other laboratory parameters—including lactate and coagulation profiles—were within normal limits. Abdominal CT revealed sigmoid volvulus with PI (Figure 1A). Despite the recommendation for immediate surgical treatment, the patient and their family members declined. As an alternative, conservative management strategies, including gastrointestinal decompression and anti-infection therapy, were promptly initiated. Three days later, the patient’s condition continued to deteriorate. Subsequently, enteroscopy confirmed MC and sigmoid necrosis (Figures 1B,C). After recommunication, informed consent for surgical intervention was obtained, and a sigmoidectomy with volvulus reduction and descending colostomy was performed. Intraoperative findings included a massively dilated sigmoid colon with a 270-degree volvulus. The intestinal wall appeared thickened, edematous, and necrotic, with extensive intramural air. The mesentery was congested, necrotic, and foul-smelling, and purulent-hemorrhagic ascites were present in the abdominal and pelvic cavities.

**Figure 2:**
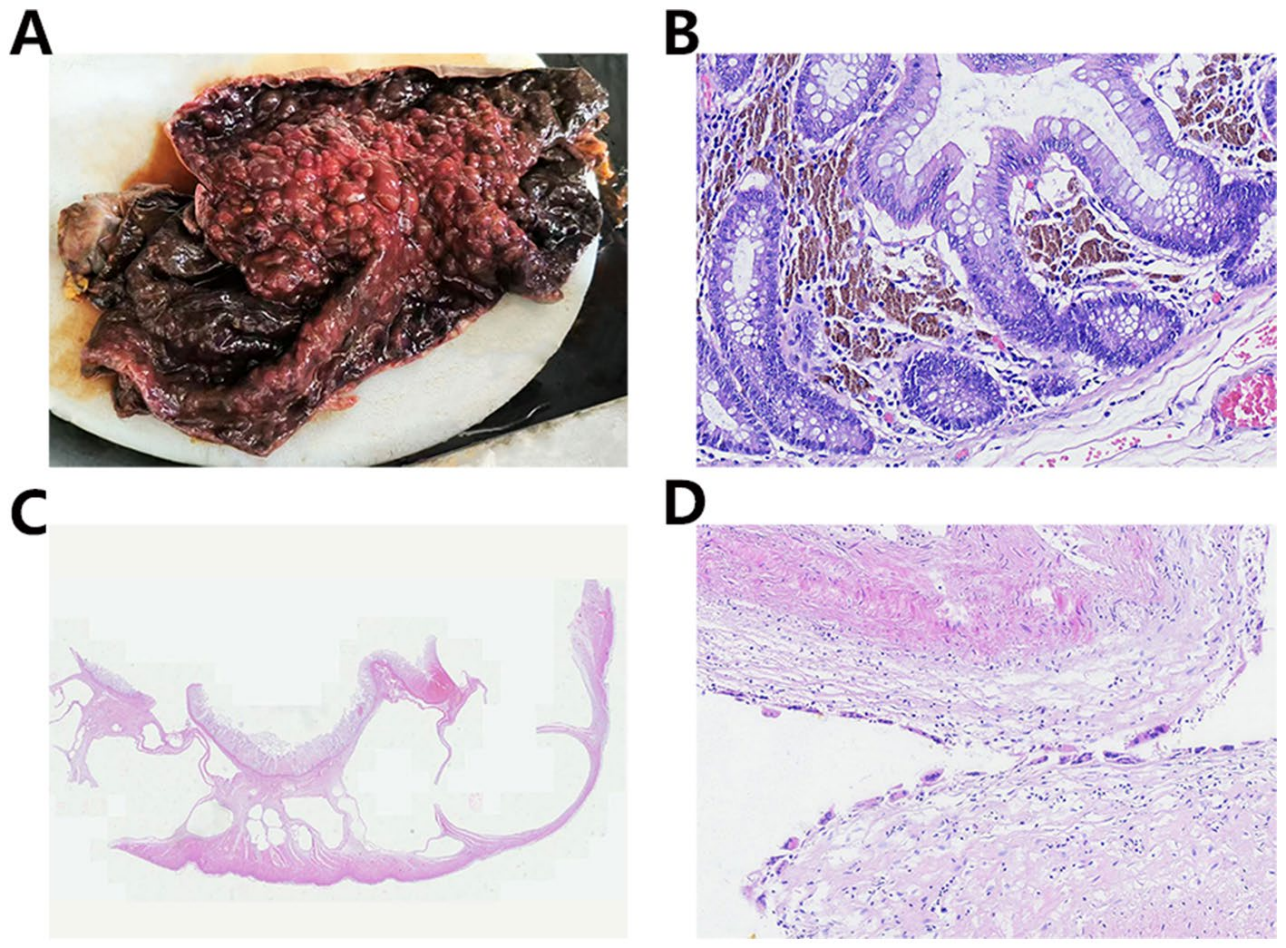
**(A)** Gross examination shows that the mucosa was partially black-brown with a cobblestone-like appearance. **(B)** The lamina propria contains abundant deposits of lipofuscin pigment. **(C)** Numerous variably sized cysts were observed, extending from the submucosa to the serosa. **(D)** Histiocytic and multinucleated giant cell reactions were noted within the cyst walls.

Upon gross examination, the resected sigmoid colon measured 60 cm in length, with a diameter ranging from 10 to 18 cm. The bowel exhibited diffuse necrosis and appeared purplish-black, and the lumen was impacted by fecal material. No discernible masses or ulcerations were observed. The intestinal wall showed irregular thickening and thinning, with the thinnest segment measuring approximately 0.2 cm in thickness. The mucosa in this thinned region appeared black-brown and flattened, with a loss of mucosal folds. In contrast, the thickened regions displayed grey-red or partially black-brown mucosa with a cobblestone-like appearance, characterized by densely arranged hemispherical protrusions (0.5–1 cm diameter) (Figure 2A). On the cut section, the bowel wall revealed a honeycomb pattern formed by multiple variably sized cysts.

**Figure 3:**
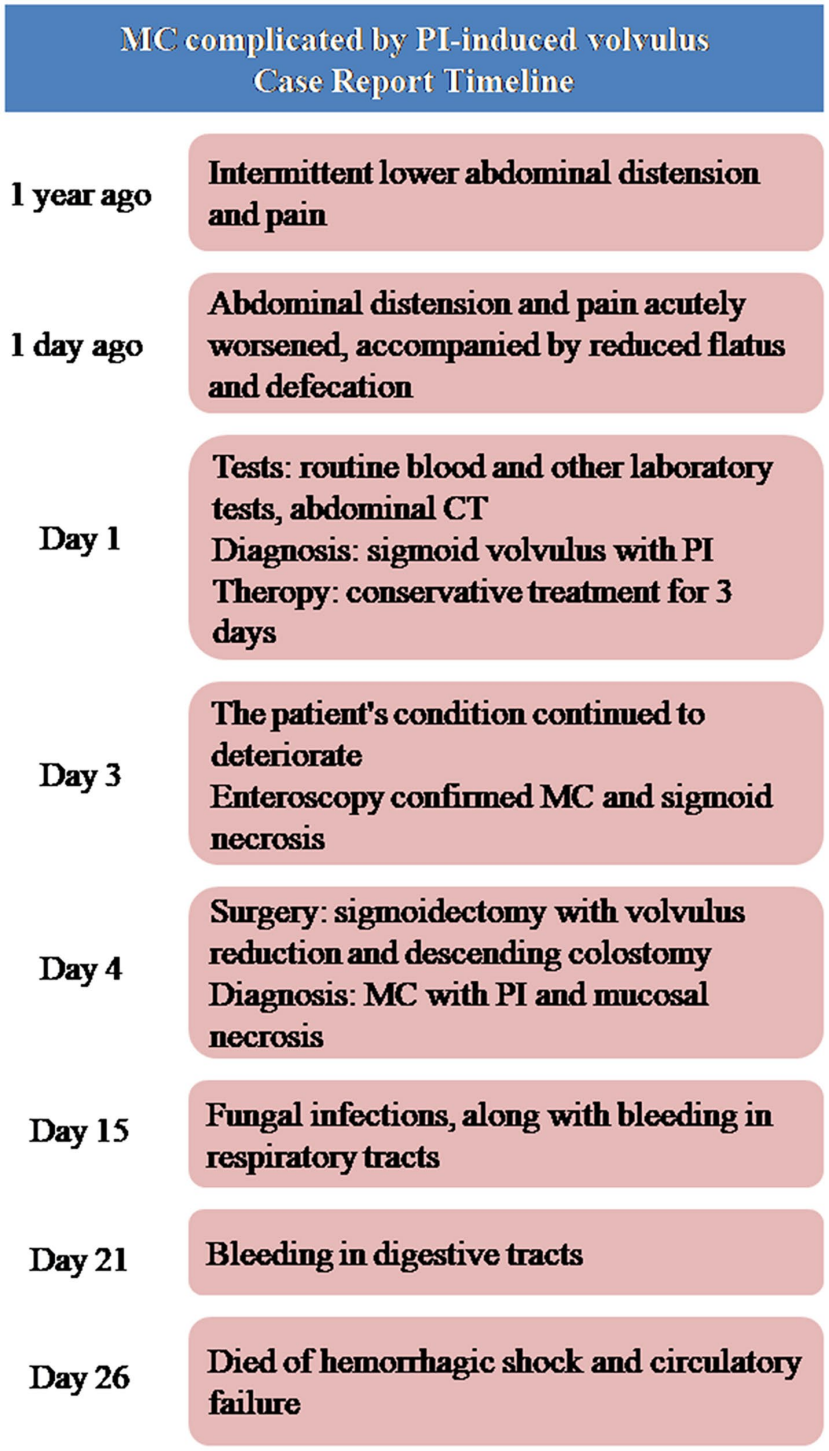
The timeline of the case.

Microscopic examination revealed extensive mucosal necrosis. The lamina propria contained abundant deposits of lipofuscin pigment (Figure 2B). In the thickened regions, numerous variably sized cysts were observed, extending from the submucosa to the serosa (Figure 2C). These cysts lacked an epithelial lining, although focal histiocytic and multinucleated giant cell reactions were observed within the cyst walls (Figure 2D). Based on these findings, the diagnosis of MC with PI and mucosal necrosis was confirmed. Postoperatively, the patient developed fungal infections along with bleeding in both the digestive and respiratory tracts. Despite intensive treatment, the condition rapidly deteriorated, leading to hemorrhagic shock, followed by circulatory failure, and the patient eventually died 22 days after surgery. The progress and decision-making processes involved in this case are shown in the timeline (Figure 3).

## 3 Discussion

The co-occurrence of MC and PI is not accidental but stems from intricate pathophysiological correlations, as substantiated by clinical observations and relevant literature. These correlations are specifically manifested in the following three key aspects. First, constipation is a common risk factor for both diseases. On the one hand, constipation promotes the abuse of anthraquinone laxatives, the primary cause of MC. On the other hand, accumulated feces in the intestine lead to chronic intraluminal pressure elevation, a persistent high-pressure state that provides the necessary mechanical conditions for PI formation. Furthermore, anthraquinone laxatives damage colonic epithelial cells and disrupt the mucosal barrier through direct injury or induction of oxidative stress, thereby establishing conditions that facilitate bacterial invasion of the intestinal wall. Finally, in the late stage of MC, the intestinal wall often exhibits thinning and smooth muscle atrophy, leading to reduced mechanical strength, which facilitates gas penetration through the mucosal barrier and promotes PI formation (8, 9). In the present case, gross examination confirmed that the mucosa in the thinned region appeared black-brown and flattened with loss of mucosal folds—a pathological feature consistent with advanced MC.

**Figure 4:**
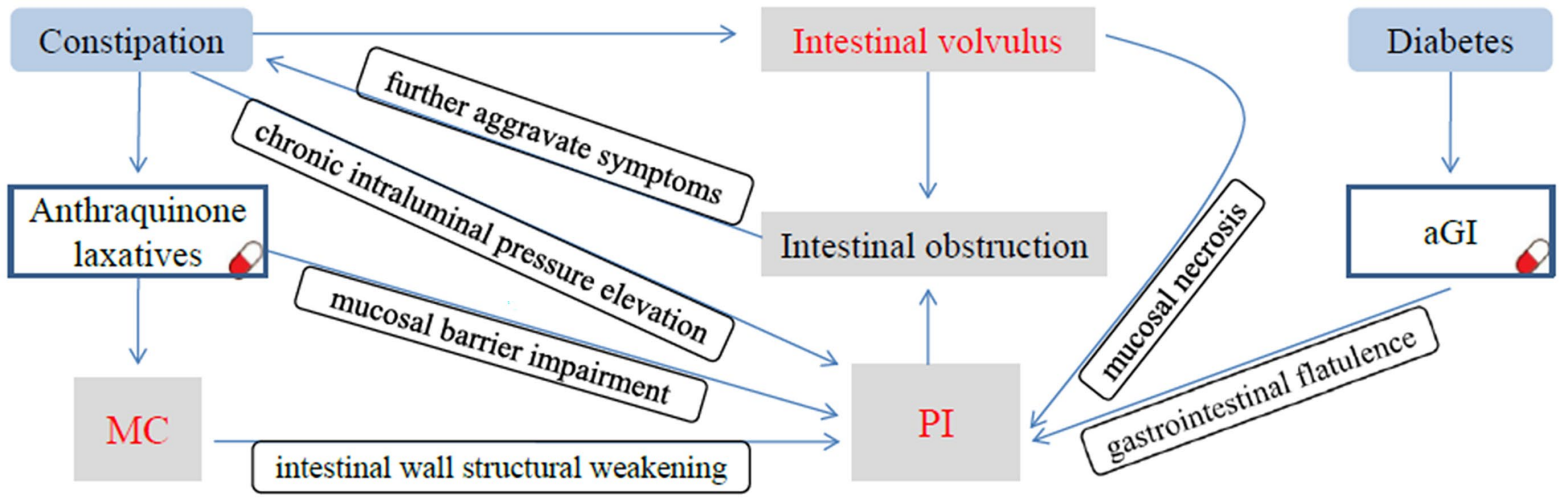
The causal relationship diagram of MC, PI, and intestinal volvulus. Chronic constipation and long-term use of anthraquinone-containing laxatives not only induce MC but also lead to persistent elevation of intraluminal pressure, causing mucosal barrier impairment and structural weakening of the intestinal wall. Meanwhile, long-term administration of αGIs results in excessive gas production in the intestinal lumen. These factors collectively induce PI. Following the onset of PI, incomplete intestinal obstruction occurred, which further exacerbated the patient’s chronic constipation. As the condition progressed, severe constipation ultimately induced the sigmoid volvulus. The complete intestinal obstruction caused by volvulus not only further worsens constipation but also leads to mucosal ischemia and necrosis, making it easier for intestinal gas to penetrate the intestinal wall and further aggravating the preexisting PI.

PI has also been documented as a rare adverse effect of alpha-glucosidase inhibitors (αGIs) therapy in diabetes management. A recent analysis by the US Food and Drug Administration Adverse Event Reporting System demonstrated a significant association between *α*GI use and PI, with diabetic patients receiving αGI exhibiting a higher incidence of PI (10). αGI competitively inhibits the digestion and absorption of starch and oligosaccharides by α-glucosidase and other digestive enzymes located on the brush border of the small intestinal mucosa, thereby reducing the postprandial blood glucose levels in humans. Undigested and unabsorbed carbohydrates are subsequently fermented by intestinal bacteria to produce hydrogen, methane, and carbon dioxide, which can lead to gastrointestinal flatulence and further induce PI. PI induced by αGIs predominantly occurs in older diabetic patients with cardiovascular diseases (6). The present case involved an 85-year-old man with a 10-year history of hypertensive heart disease and a 6-year history of poorly controlled type 2 diabetes—characteristics that align with this high-risk population. He had been taking acarbose (an αGI) long-term for glycemic control, whereas concurrent prolonged use of *Binglang Sixiao Pills* (an anthraquinone-containing laxative) had already induced MC and intestinal mucosal damage. This “dual-hit” scenario significantly elevated the risk of developing PI. We hypothesize that following the onset of PI, incomplete intestinal obstruction may have occurred, which further exacerbated the patient’s chronic constipation. Over time, severe constipation (a well-documented risk factor for sigmoid volvulus in elderly patients) ultimately induced sigmoid volvulus, and subsequent complete intestinal obstruction from the volvulus not only worsened constipation but also impaired intestinal blood supply, leading to progressive mucosal ischemia and necrosis. These pathological changes, including exacerbated mucosal damage and increased gas penetration through the compromised intestinal wall, subsequently aggravated the preexisting PI, forming a vicious cycle characterized by mutual exacerbation between the volvulus and PI. A causal relationship diagram of MC, PI, and intestinal volvulus is presented in Figure 4.

The primary treatment for MC involves discontinuing anthraquinone-containing compounds. Resolution of the condition may take several months to a year. Alternative therapies for constipation that do not induce mucosal pigmentation should be considered for patients with MC, including osmotic laxatives, fiber supplements, and secretagogues (11). The treatment of PI depends on the underlying causes and associated complications. The primary etiology must be addressed before initiating specific treatments. For asymptomatic patients or those with mild symptoms, conservative management is sufficient, including hyperbaric oxygen therapy, antibiotics to suppress intestinal bacterial infections, and endoscopic intervention. However, approximately 3% of patients with PI develop severe complications, such as intestinal perforation, obstruction, or ischemia, necessitating prompt surgical intervention. Emergency exploratory laparotomy should be considered in patients presenting with signs of peritonitis, metabolic acidosis, lactate levels > 2.0 mmol/L, or portal venous gas (12). The death of this patient resulted from the superimposition of multiple factors. First, the direct cause of death was a cascade of multiple organ failures. The intraoperative findings of diffuse necrosis of the sigmoid colon, congestive necrosis of the mesentery, and purulent hemorrhagic ascites in the abdominal cavity indicated severe disruption of the intestinal barrier. Many intestinal pathogenic bacteria invaded the abdominal cavity through the damaged intestinal wall, causing severe peritonitis. The infection rapidly progressed to septic shock, leading to peripheral vasodilation and insufficient tissue perfusion, which further induced multiple organ dysfunction syndrome (MODS). Gastrointestinal bleeding was directly related to intestinal necrosis, mucosal ulcers, and coagulation dysfunction; respiratory bleeding might be caused by pulmonary microvascular injury, or alveolar hemorrhage induced by septic shock; and the fungal infection that occurred after surgery further weakened the body’s immune function and exacerbated organ damage. Second, the superimposition of underlying diseases amplified the risks. An 85-year-old patient had long-term diabetes with poor blood glucose control, which had already led to vascular lesions and immune dysfunction. Meanwhile, the concurrent hypertensive heart disease reduced cardiac reserve function, making it difficult for patients to tolerate the circulatory load caused by septic shock. These underlying conditions collectively constituted potential hidden dangers for the deterioration of the patient’s condition. Third, delayed treatment aggravated irreversible damage. The patient and his family initially refused to undergo surgery. During the 3-day conservative treatment period, the intestinal volvulus was not relieved, and ischemic necrosis of the intestinal wall continued to progress, missing the optimal intervention opportunity. Although surgical resection of the diseased intestinal segment was performed later, the scope of the intestinal necrosis had expanded significantly by then, and systemic infection and organ damage had entered an irreversible stage, ultimately making it impossible to reverse the patient’s condition. For analogous cases, the following targeted interventions should be implemented to optimize outcomes. First, expedited surgical intervention is critical. Concomitantly, enhancing risk communication during the informed consent process is essential, with a specific emphasis on the hazards of conservative management in cases where the sigmoid volvulus is complicated by PI. Prolonged obstruction exacerbates MC-associated mucosal damage and intramural gas accumulation, which elevates the risk of life-threatening complications such as hemorrhagic shock. Second, the proactive management of high-risk medication interactions is warranted. Given the synergistic risks posed by anthraquinones (which disrupt the mucosal barrier) and αGIs (which promote intraluminal gas accumulation via carbohydrate fermentation), immediate discontinuation of anthraquinone laxatives upon admission is prioritized, accompanied by temporary suspension of αGIs. These measures may mitigate PI progression prior to surgical intervention. Finally, enhanced perioperative infection control strategies are imperative. The fatal septic shock observed in this case resulted from intestinal barrier compromise. For similar patients, preoperative initiation of broad-spectrum antibiotics covering Gram-negative and anaerobic pathogens is recommended to limit infection dissemination. Regarding preventability, although the patient’s advanced age, multiple comorbidities, and late presentation conferred substantial baseline risks, earlier surgical intervention and targeted medication adjustments might have altered the outcome. However, the convergence of MC-related mucosal fragility, PI-induced intramural gas, and volvulus-associated ischemia creates an inherently high-risk scenario in which even optimal management cannot guarantee survival—highlighting the critical need for proactive risk stratification in this vulnerable population.

## 4 Conclusion

In conclusion, we report the case of an elderly patient with MC complicated by PI that resulted in intestinal volvulus and necrosis. The patient had a history of chronic constipation, hypertensive heart disease, and diabetes mellitus. While the independent pathogenesis of MC and PI is relatively well established, their coexistence is exceptionally rare. We hypothesize that the use of αGI to reduce postprandial blood glucose levels may contribute to intestinal gas accumulation. Concurrently, the use of anthraquinone-containing laxatives likely causes intestinal mucosal damage and increased permeability. This not only leads to the characteristic pigment deposition of MC (formed by macrophages phagocytosing apoptotic cells) but may also permit intestinal gas to penetrate the intestinal wall through compromised mucosa, thereby forming PI. The following conclusions can be drawn from this study: First, enhanced monitoring of high-risk populations is imperative. Elderly diabetic patients with chronic constipation and underlying cardiovascular diseases who are concurrently using anthraquinone-containing laxatives and αGIs have a significantly elevated risk of developing PI. Regular abdominal CT or endoscopic screening should be performed to detect PI and intestinal mucosal lesions, avoiding intervention only after the onset of acute symptoms. Second, for patients with PI complicated by acute intestinal volvulus, the high risks of conservative treatment (such as intestinal necrosis and infection spread) must be fully communicated, and efforts should be made to perform surgery as early as possible to relieve obstruction. Third, enhanced education for both healthcare professionals and patients regarding the rational use of natural laxatives is essential. Chronic high-dose use to alleviate symptoms should be avoided. For adult constipation management, the short-term use of anthraquinones should be limited to ≤ 30 mg/day, administered 2–3 times weekly. Any treatment extending beyond 1–2 weeks requires medical supervision (3). The fatal outcome of this case not only reflects the complexity of the disease itself but also highlights the need for improvement in clinical practice regarding the identification of high-risk populations and the timeliness of treatment decisions, providing important practical evidence for reducing the mortality rate of similar cases in the future.

## Data Availability

The original contributions presented in the study are included in the article/supplementary material, further inquiries can be directed to the corresponding author.
